# Mechanical Phenotyping of MG63s Following Vibrational Stimulation

**DOI:** 10.1096/fj.202504885R

**Published:** 2026-05-02

**Authors:** Rui Pedro Pereira Sousa, Stuart Reid, Matthew J. Dalby, Melanie Jimenez, Peter G. Childs

**Affiliations:** ^1^ Centre for Cellular Microenvironment, Department of Biomedical Engineering University of Strathclyde Glasgow UK; ^2^ Wolfson Centre Glasgow UK; ^3^ Centre for the Cellular Microenvironment, Institute of Molecular Cell and Systems Biology University of Glasgow Glasgow UK

**Keywords:** actin, cytoskeleton, Mechanotransduction, MG63, Nanovibration

## Abstract

Nanovibration, a kilohertz‐frequency, nano‐amplitude mechanical stimulation, has been shown to drive osteogenesis; however, the mechanisms remain unclear. Mechanotransduction has been proposed with limited cell population‐level evidence. We propose the use of a high‐throughput mechanical phenotyping technique, Real‐Time Deformability Cytometry (RT‐DC), to observe mechanical changes in an osteogenic model, MG63s. We have demonstrated that MG63 cells respond to the nanovibrational mechanical stimulation by changing their cytoskeletal morphology and showing higher expression of osteocalcin than respective controls. We have also demonstrated the first use of Real‐Time Deformability Cytometry (RT‐DC) to reliably phenotype whole‐cell population mechanical response to this stimulus. The use of high‐throughput microfluidic techniques such as RT‐DC is proving invaluable to more accurately assay population morphological changes compared to other established techniques, with potential application in mechanobiology, cellular quality control, and diagnostic scenarios. With consideration for osteogenic changes, RT‐DC also poses potential use in the assessment of in vitro and ex vivo bone cell samples, highlighting clinical relevance for conditions such as osteoporosis and bone fracture.

## Graphical abstract

Mechanical Phenotyping of MG63s Following Vibrational Stimulation, *Mr. Rui Pedro Pereira Sousa, Prof Stuart Reid, Prof Matthew J. Dalby, Dr. Melanie Jimenez and *Dr. Peter G. Childs. Summary of the use of a high throughput mechanical phenotyping technique, Real‐Time Deformability Cytometry (RT‐DC) to observe mechanical changes in an osteogenic model, MG63s. This study establishes RT‐DC as a powerful tool for mechanobiology research, as well as further showing it is potential for cell engineering and cellular diagnostics.

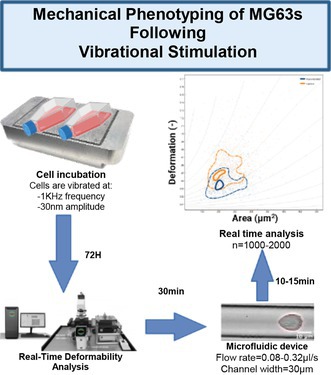

## 1 Introduction

Bone mineral density (BMD) loss presents a significant challenge particularly in an aging population, increasing the risk of injury or fractures [1]. However, although osteoporosis is already a well‐recognized and studied problem of decreased BMD reported to affect more than 27.5 million people in the European Union (EU) alone [2], the underlying cause, the imbalance between bone formation and resorption [3] must be studied further. In a healthy individual, bone is constantly self‐renewing [4]. This process is conducted by two cell types: osteoblasts and osteoclasts [5]. Osteoblasts are responsible for bone formation, whereas osteoclasts lead bone resorption [4].

Current treatments consist of medication designed to slow down or freeze the disease advancement, commonly consisting of antiresorptive agents such as bisphosphonates or anabolic agents such as Teriparatide and Romosozumab [6, 7, 8, 9]. Although these drugs are capable of stimulating bone formation or stopping its resorption, they also exhibit a range of side effects ranging from gastrointestinal irritation to osteonecrosis of the jaw, among other complications [10, 11, 12]. Limitations such as this have driven interest in researching drug‐free approaches to mechanically modulate the bone cell's behavior.

Mechanical stimulation has been studied as a potential drug‐free treatment option that focuses on the use of nano‐amplitude, kHz frequency vibrations to promote bone cell differentiation [13, 14, 15] while reducing osteoclast formation given the correct conditions [16]. However, the mechanistic basis of this response remains poorly understood. Nanovibration‐induced osteogenesis is likely a mechanotransducive phenomenon; however, data have been inconsistent. The MG63 osteosarcoma cell line was chosen as it serves as a robust model for osteogenesis due to its stable phenotype and expression of key osteogenic markers such as osteocalcin [17]. Mechanically, a study conducted by Docheva et al. has found no statistically significant changes in the stiffness of human primary osteoblasts and MG63s [18]. When responding to a stimulus, cytoD, a disruptor of F‐actin assembly, although MG63s presented initially with less nuclear YAP, they presented the same nuclear YAP decrease response as osteoblasts, showing the MG63s sensitivity for mechanotransduction [19].

It is currently unknown how phenotype and cellular mechanical properties are related under this type of vibrational stimulation. Current methods, such as Atomic Force Microscopy (AFM), struggle with low throughput; for instance, a 64 × 64‐pixel modulus map, with one indentation per second, would take more than an hour. Even fast force mapping modes, which are taken at 512 × 512 pixels in the kHz range, will take several minutes to map one sample [20]. In contrast, Real‐Time Deformability Cytometry (RT‐DC) is capable of analyzing up to one thousand cells per second when in suspension, depending on sample concentration and flow rate [21]. A microfluidic approach to cellular mechanical testing allows for high‐throughput quantification, thus making it more reliable to gauge population response to a stimulus [22, 23]. Furthermore, during testing, pillars in the inlet of the microfluidic device filter out clumps of cells. Due to the nature of image cytometry, all measured cells are indexed to an image, which can be qualitatively checked for cell integrity and ensure the equipment is properly measuring single well‐defined cells. In the same fashion as AFM, where attention needs to be taken when selecting the correct probe, the same attention needs to be taken when selecting the microfluidic device's channel size. Channel size is important as the cell size can influence the amount of shear force that the cells experience [24]. The technique will, however, lack the resolution to distinguish the stiffness from the nucleus in comparison to the cytoplasm; however, AFM cannot always disentangle these either. Unlike AFM, where cells are measured in adherence, RT‐DC involves detaching cells for measurement.

Here, we demonstrate that mechanical changes, induced by nanovibration, are still measurable for detached cells, making this a potential option for high‐throughput fingerprinting of cellular mechanical response. RT‐DC will not substitute AFM, but its potential to become a more appropriate screening tool for mechanobiology will be investigated in this paper.

## 2 Methodology

### 2.1 Cell Culture

Osteosarcoma immortalized cells, MG63, were cultured in T75 cell culture flasks (Fisher Scientific, 10 364 131) using Dulbecco's Modified Eagle Medium (DMEM) (VWR, 392–0415) supplemented with 10% (v/v) fetal bovine serum (FBS) (Fisher Scientific, 17 593 595), non‐essential amino acids (Thermo Fisher, 11 140 050), and Penicillin–Streptomycin (Pen/Strep) (Merck, P4458) in a humidified incubator at 37°C in 5% CO_2_. Cells from the same batch were passaged at 80% confluence, being split into fresh cell culture flasks using ethylenediaminetetraacetic acid (EDTA) (Thermo Fisher, 17 892) and 0.05% (w/v) Trypsin (Merck, T4674).

### 2.2 Vibrational Stimulation of MG63s

**FIGURE 1:**
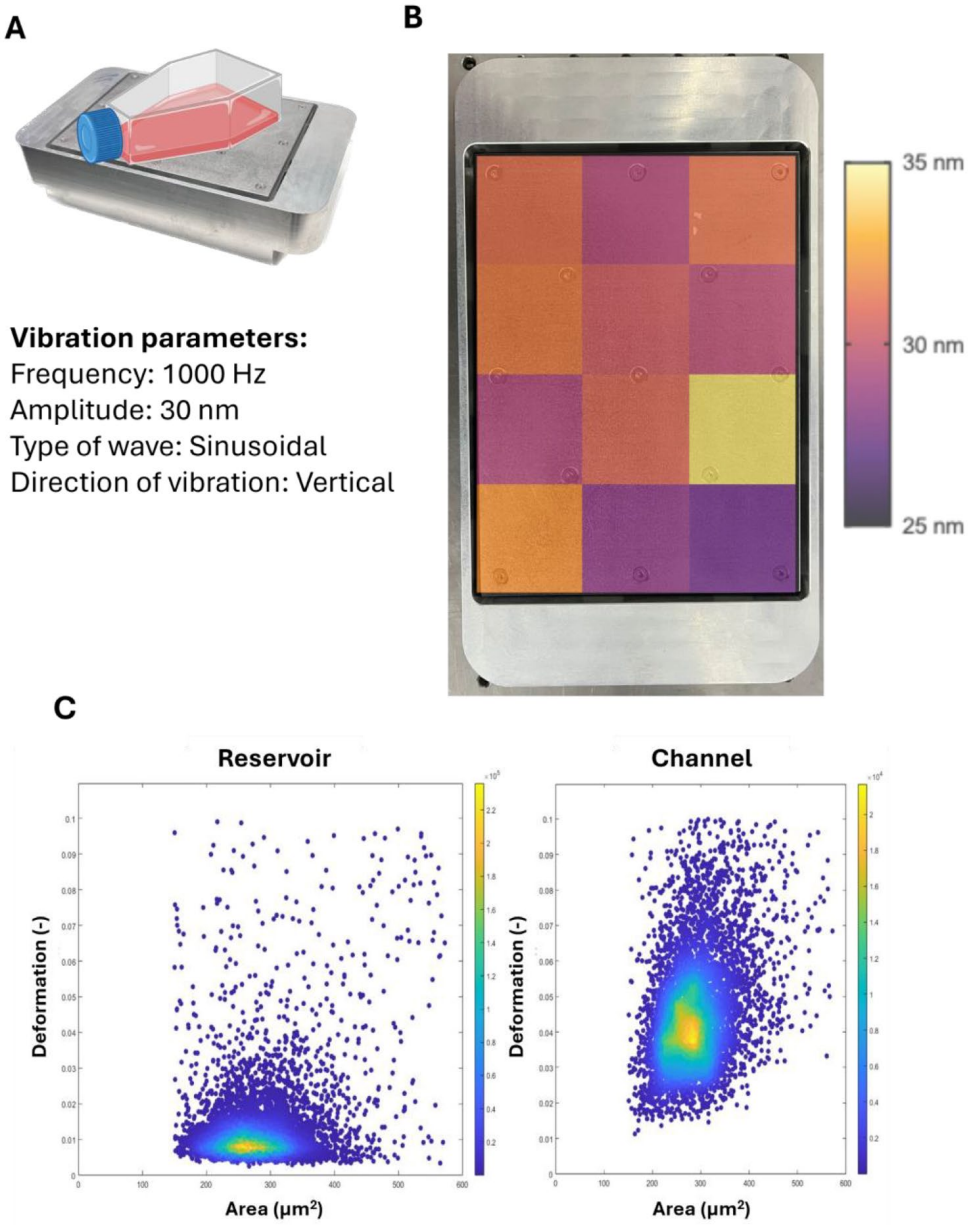
Mechanical stimulation setup and high‐throughput whole cell mechanical analysis. (A) T75 flasks are attached to the nanovibration device by adhesive magnets. The device utilizes piezo actuators to vibrate the plate vertically. The nanovibration device is calibrated to output sinusoidal motion of 1000‐Hz frequency and 30‐nm amplitude. (B) Bioreactor's calibration profile using laser interferometry presenting an output signal of 1000 Hz frequency and 30.42 ± 1.75 nm (Mean ± SD) amplitude. (C) Cell's mechanical properties are tested utilizing Real‐Time Deformability Cytometry. The samples are tested under zero stress (reservoir) and under stress (channel). High‐throughput technique allows testing *n* = 5000 under 2 min assuming 1 × 10^6^ cells/mL.

The bespoke nanovibration device used to mechanically stimulate the osteosarcoma cell line is described in Campsie et al. (2019) [14] but briefly is constituted of an aluminum block housing an array of piezo actuators that, in turn, support a ferrous contact plate (Figure 1A). The aluminum block provides counter mass in order to direct vibration of the piezo actuators upward to the contact plate. Tissue culture plates are bonded to self‐adhesive magnetic sheets (3 M), allowing temporary, conformal attachment to the contact plate. This allows plates to be removed for routine testing or changes in media. Nanovibration devices were calibrated via laser interferometry to output a sinusoidal vibration of 30‐nm amplitude by modulating the drive voltage provided to the piezo actuators. The vibration frequency is determined by the power supply and is set to 1000 Hz for all experiments. Cells were cultured and undisturbed for 24 h prior to stimulation to allow attachment. Vibrational stimulation was initiated at *t* = 0 h.

### 2.3 Real‐Time Deformability Cytometry

Detachment of cells from cultureware was performed using 0.05% (w/v) trypsin preceded by two washing steps of EDTA (Thermo Fisher, 17 892). Trypsinised cells were diluted in DMEM cell media (VWR, 392–0415) at a 1:5 ratio. Cell concentration was adjusted with DMEM cell media to reach 3 × 10^5^ cells/mL in 5 mL of the sample. The 5 mL sample was centrifuged at 800 RCF for 5 min. The supernatant was removed, and cells were resuspended in 1 mL of Cell carrier B (ZellMechanik Dresden, ZM‐C‐CC‐049) at a 1.5 × 10^6^ cells/mL concentration. The cell's deformability measurements were conducted in the PDMS 30 μm channel chip (ZellMechanik Dresden, ZM‐C‐FXX) at flow rates 0.08, 0.12, 0.16, and 0.32 μL/s on both channel and reservoir. All measurements were performed 20 min following flask's trypsinization.

### 2.4 MTT Assay (Proliferation Analysis)

MTT assay was performed utilizing Abcam's MTT Cell Proliferation Assay Kit (Abcam, Ab211091). The protocol utilized was version 2b (last updated 05 June 2023) of the same kit.

### 2.5 Permeabilization Buffer

Permeabilization buffer is formulated using Phosphate Buffered Solution (PBS) (VWR, 392–0442) and adding 10.3 mg/mL sucrose (Merck, S9378), 0.292 mg/mL sodium chloride (Merck, S7653), 0.060 mg/mL magnesium chloride (Merck, M8266), and 0.476 mg/mL 4‐(2‐hydroxyethyl)‐1‐piperazineethanesulfonic acid (HEPES) (Merck, H3375). The pH was adjusted with hydrochloric acid (Thermo, 123 456) and supplemented with 0.5% (v/v) TritonX (Merck, X100).

### 2.6 Immunocytochemistry

Cells were fixed utilizing 10% buffered formalin (VWR, 11699455) and washed in PBS (VWR, 392–0442). Samples were incubated for 30 min at 4°C in permeabilization buffer and blocked in 1% (w/v) Bovine Serum Albumin (BSA) (Merck, A9418) in PBS at 37°C for 7 h. Primary OCN antibody (Abcam, ab13361) was diluted at 1:100 in 1% (w/v) BSA in PBS and incubated in the sample for 16 h at 4°C. The antibody was removed and washed in 0.5% (v/v) Tween (Thermo Scientific, J63596.AP) in PBS. Following the washes, TxRed secondary antibody (Abcam, ab6719) was diluted in 1% BSA in PBS, added to the sample, and incubated for 2 h at 37°C. The antibody was removed, and the sample was washed in 0.5% (v/v) Tween in PBS. Phalloidin (Abcam, ab235137) was diluted 1:1000 in 1% (w/v) BSA in PBS, added to the sample, and incubated at room temperature for 1 h. The samples were rinsed in PBS and stained with 300 nM DAPI (Merck, D9564). DAPI was removed, and the sample was washed in PBS and preserved in PBS at 4°C for short‐/medium‐term storage. Fluorescent staining analysis was performed utilizing ImageJ version 1.53 t.

### 2.7 Fluorescent Intensity Quantification

Using ImageJ version 1.53 t, quantification of actin and OCN stains was performed using the Corrected Total Cellular Fluorescence (CTCF) method described by McCloy et al. (2014) [25].

### 2.8 Actin Heterogeneity

ImageJ version 1.53 t was utilized to draw a profile of the actin intensity at the transects lines of the cells in their minor axis: above the nucleus, on the nucleus, and below the nucleus. Actin “roughness” or heterogeneity was calculated by calculating the average deviation of the actin intensity peaks of the profile from the average intensity along the profile [26].

### 2.9 Statistical Testing

Statistical analysis of data was conducted using GraphPad Prism 10 (version 10.0.3 (217)). The statistical test conducted was a Kruskal–Wallis test as data was non‐normally distributed and prevenient from more than two groups. Dunn–Bonferroni *p*‐value adjustment was performed to account for the number of pairs being compared. All comparisons between control and treated groups when conducted at different time points, that is, CT versus NV where 0 h is present, also were compared against each other using the nonparametric test equivalent to Kruskal–Wallis for two samples, the Mann–Whitney test. All statistical data displayed on figures are Kruskal–Wallis tests unless otherwise stated. MTT statistical analysis was conducted using a two‐way ANOVA.

## 3 Results

### 3.1 Mechanically Treating and Testing MG63s

Mechanical treatment was applied to the cell as represented in Figure 1A. The device was calibrated using laser interferometry averaging a 30.42‐nm vertical displacement at 1000 Hz across the plate with a 5.74% coefficient of variation (Figure 1B). Mechanical phenotyping was achieved using the high‐throughput Real‐Time Deformability Cytometry (RT‐DC) technique. This approach to mechanical testing yields large datasets as seen in Figure 1C. Furthermore, the data also grant the user images of all cells represented in the graph, allowing for visual quality control of data and physical associated features to trends present.

## 4 RT‐DC Parameter Optimization

**FIGURE 2:**
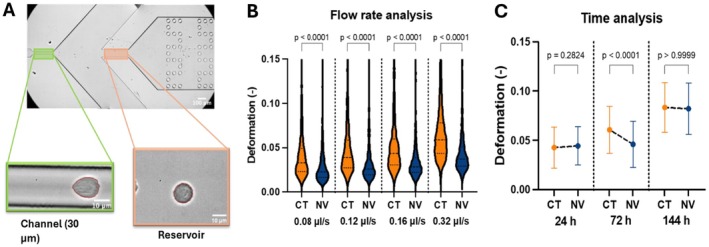
Demonstration, testing, and optimization of mechanical stimulation and characterization of MG63 cells. (A) RT‐DC 30 μm PDMS chip utilized to test cells. Cells tested on reservoir and channel. Reservoir measures 100 μm wide where cells are not submitted to compressive stresses and therefore demonstrate the cells' natural relaxed state. Cells measured in the channel presented conformance to a bullet shape. The shape in the channel dictates the deformation values. (B) The flow rate also will increase or decrease the compressive forces in the channel allowing us to obtain a better resolution. The flow rate of 0.32 μL/s was the highest possible flow rate due to equipment technical limitations and presented itself as the optimal flow rate to test the cells. Determining the optimal time to test the cells is also imperative. N of cells tested was 1000 per condition. Statistical testing using Mann–Whitney testing between CT and NV at each time point. (C) Cells seeded at 5000 cells/cm^2^ presented a significant statistical difference between the control and nanovibrated group at 72 h. N of cells tested was 1000 per condition. Statistical testing using Mann–Whitney testing between CT and NV at each time point. Error bars present standard deviation.

RT‐DC tests the cells in two distinct areas of interest: reservoir and channel (Figure 2A). The reservoir consists of the area where the cells are not subjected to compressive stresses and therefore present themselves in their non‐stressed state, usually spherical. Therefore, this region allows us to check whether the cells being tested in the channel are indeed strained by the stresses on them or whether it is the natural shape of the cell. As no cell is perfectly spherical, a value of deformability of zero is practically impossible. Conversely, the channel actively induces cell deformation on the cells via shear stresses caused by increased fluid pressure. This increased fluid pressure results from the lateral injection of sheath fluid at three times the flow rate of the sample fluid. It is important to remark that both sample and sheath fluids are composed of the same solution.

Assuming this 1:3 ratio, the equipment comes, by default, with three overall flow rates: 0.08, 0.12, and 0.16 μL/s. Upon consultation with the equipment's manufacturer, ZellMechanik, it was established the theoretical limitation of the equipment was 0.32 μL/s, a flow rate validated on MG63s by Herbig et al. [27]. To establish the optimal flow rate to test future samples, all four flow rates were studied (Figure 2B). To this effect, mechanically treated (NV) and non‐treated (CT) cells were incubated for 72 h and tested. Results predictively showed deformability increased for both groups of cells as the flow rate of testing increased. Most importantly, it was demonstrated that at all flow rates, the same behavior of the NV group was recorded with statistical significance, that is, the nanovibrated cells were less deformable than controls. The flow rate of 0.32 μL/s was selected as the optimal flow rate for future experiments involving MG63s as the cells present a naturally high degree of stiffness compared to the cells usually tested in this equipment such as red blood cells. Also, this flow rate allows increased strain of the cells than the lower flow rates, making differences between groups more evident. As no adverse effects were recorded from increasing the flow rate, it was selected to progress with.

With the ascertained flow rate (Figure 2B), it was important to validate the optimum testing regime. To determine the ideal time of deformability measurement, an experiment was designed to characterize mechanical changes over an early time point (24 h), intermediate time point (72 h), and a late time point (144 h) (Figure 2C). The cells were seeded at 5000 cells/cm^2^. It was noted that at 24 h (Figure 2C there was no significant difference between CT and NV groups. At 72 h (Figure 2C), the results between CT and NV replicated the same behavior as the cells presented in the flow rate experiment (Figure 2B). The NV cells were significantly stiffer than the control cells (Figure 2C). At 144 h, both groups presented with a very similar average deformability and there was no statistically significant difference between the two. The reduced difference at higher time points may be due to the confounding effect of increased cell–cell contact.

## 5 Confirming Key Variables Impacting Deformability

**FIGURE 3:**
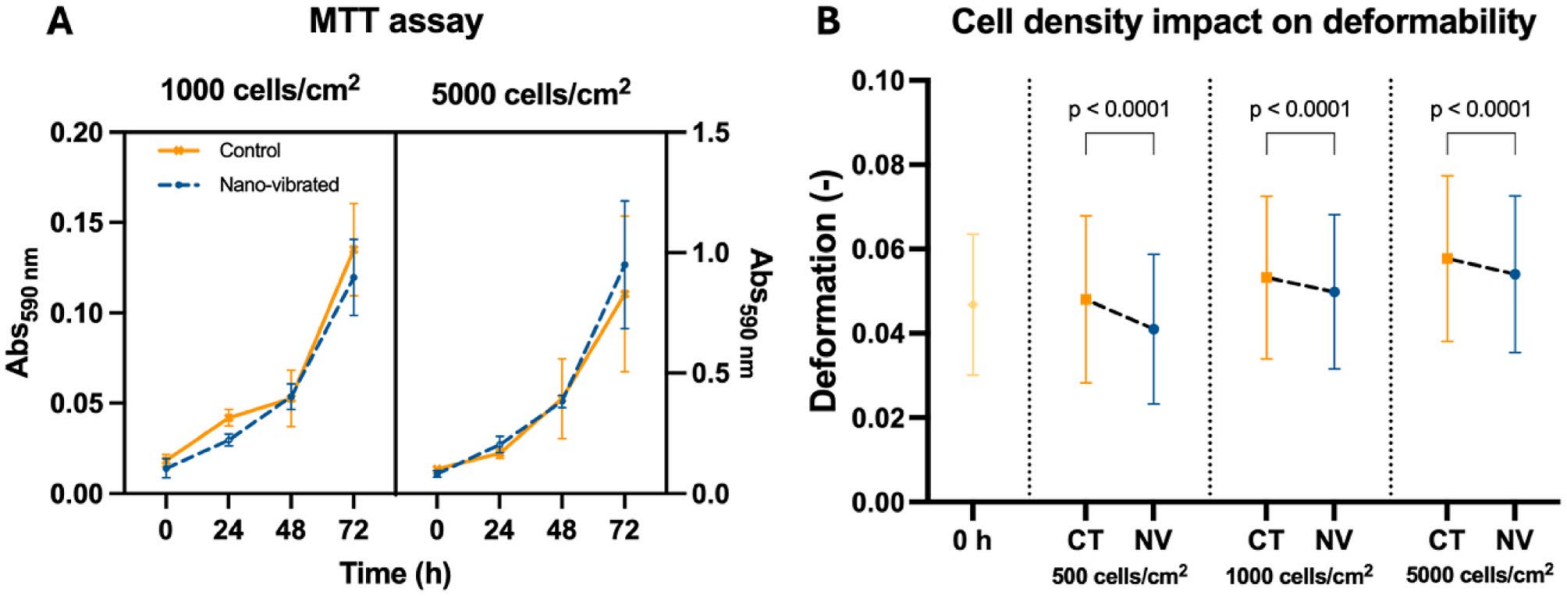
Impact of seeding density on deformability measurements. (A) Cells seeded at 1000 and 5000 cells/cm^2^ with lower seeding densities being too low to conduct RT‐DC measurements at early time points. The cells presented no differences in growth rate between treated and non‐treated groups. *N* = 3 per condition. Statistical testing performed using a 2‐way ANOVA. Error bars present standard deviation. (B) Control 0 h was seeded at 5000 cells/cm^2^ as there was too low a number of cells to conduct measurements at lower densities at such an early time point. At 72 h, the nanovibrated group presented significantly lower deformability than the correspondent controls at all seeding densities. Analyzing groups across seeding densities it is evident that as seeding density increases, so does the deformability of the cells. *N* = 5000 per condition. Statistical testing using Mann–Whitney testing between CT and NV at each seeding density. Error bars present standard deviation.

To ascertain whether there was indeed an impact on deformability originating from cell‐to‐cell interactions, varied seeding density was used to achieve confluency in a 2D culture at different time points and thereby modulate cell‐to‐cell contact. Results shown in Figure 3A demonstrate that there are no discrepancies in cell growth between seeding densities over the first 72 h. Furthermore, the testing of both CT and NV groups showed that NV does not affect the proliferation rate of the cells regardless of other potential processes the cells are undergoing (Figure 3A).

With there being no difference in proliferation rate between the seeding densities, it was necessary to analyze that the cells were responding similar to the mechanical stimulation regardless of the seeding density (Figure 3B). Results show that NV cells are consistently, significantly less deformable than the control cells at all seeding densities when measured at 72 h (Figure 3B). Furthermore, when comparing control cells at the three different seeding densities, the results show that as the density increases, the cells become significantly less stiff (*p* < 0.0001). The same trend also occurs in the NV group while maintaining a stiffer profile compared to CT (Figure 3B). Control 0 h was seeded at 5000 cells/cm^2^ as it was the only seeding density that possessed enough cells to conduct RT‐DC testing.

## 6 Mechanically Induced Expression and Cytoskeletal Changes

**FIGURE 4:**
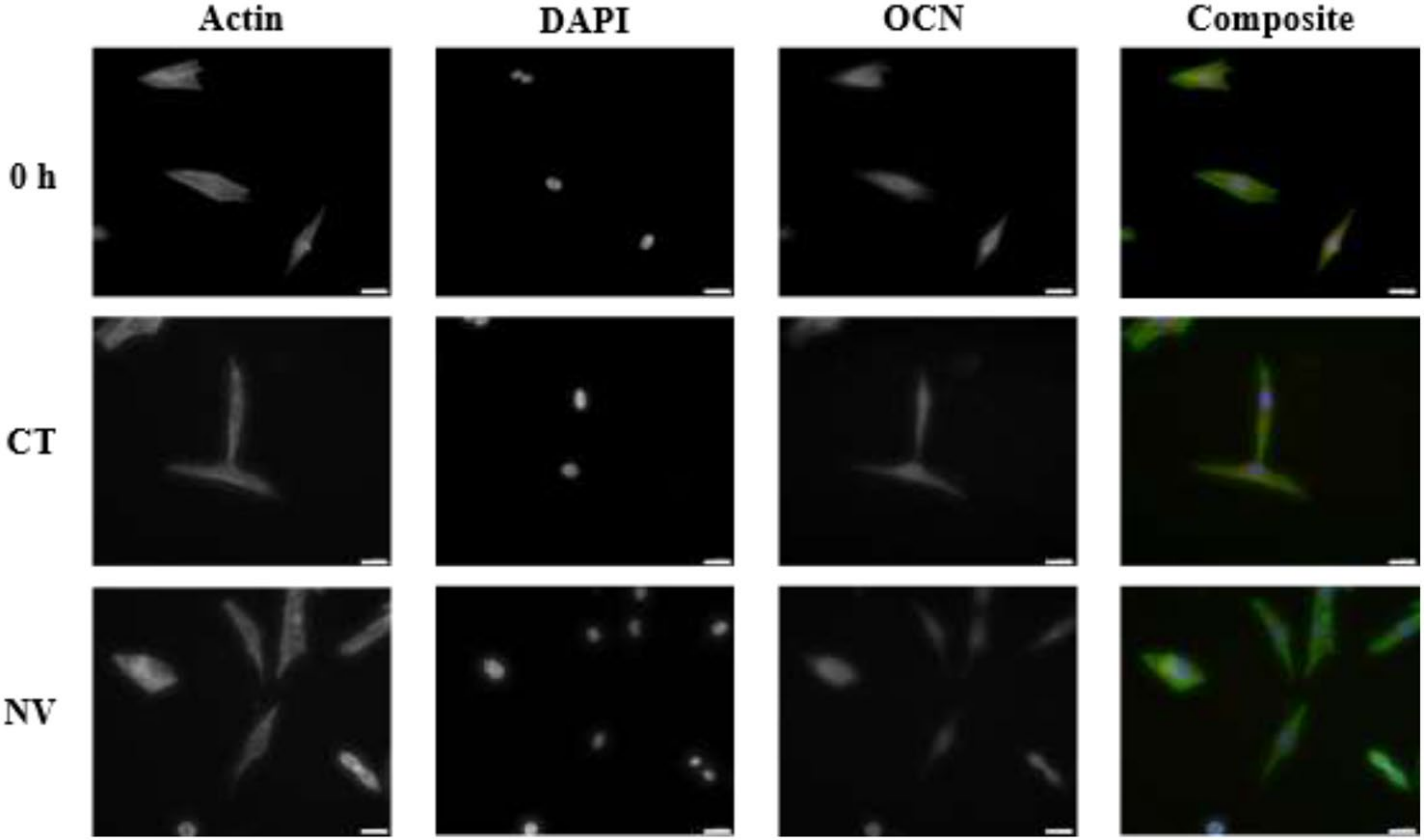
Typical immunofluorescent staining of MG63s. Phalloidin‐stained Actin (green), DAPI stained DNA (blue), and Texas‐red dyed OCN. Magnification 20×. Scale bar width is 40 μm.

Immunofluorescent staining was conducted to track morphological changes that may be affecting the cell's stiffness, as well as osteocalcin expression, which may help tie mechanical changes to osteogenic expressional changes (Figure 4). Cells were seeded at 1000 cells/cm^2^ allowing for better segmentation of the cells in the images obtained at 72 h of culture.

**FIGURE 5:**
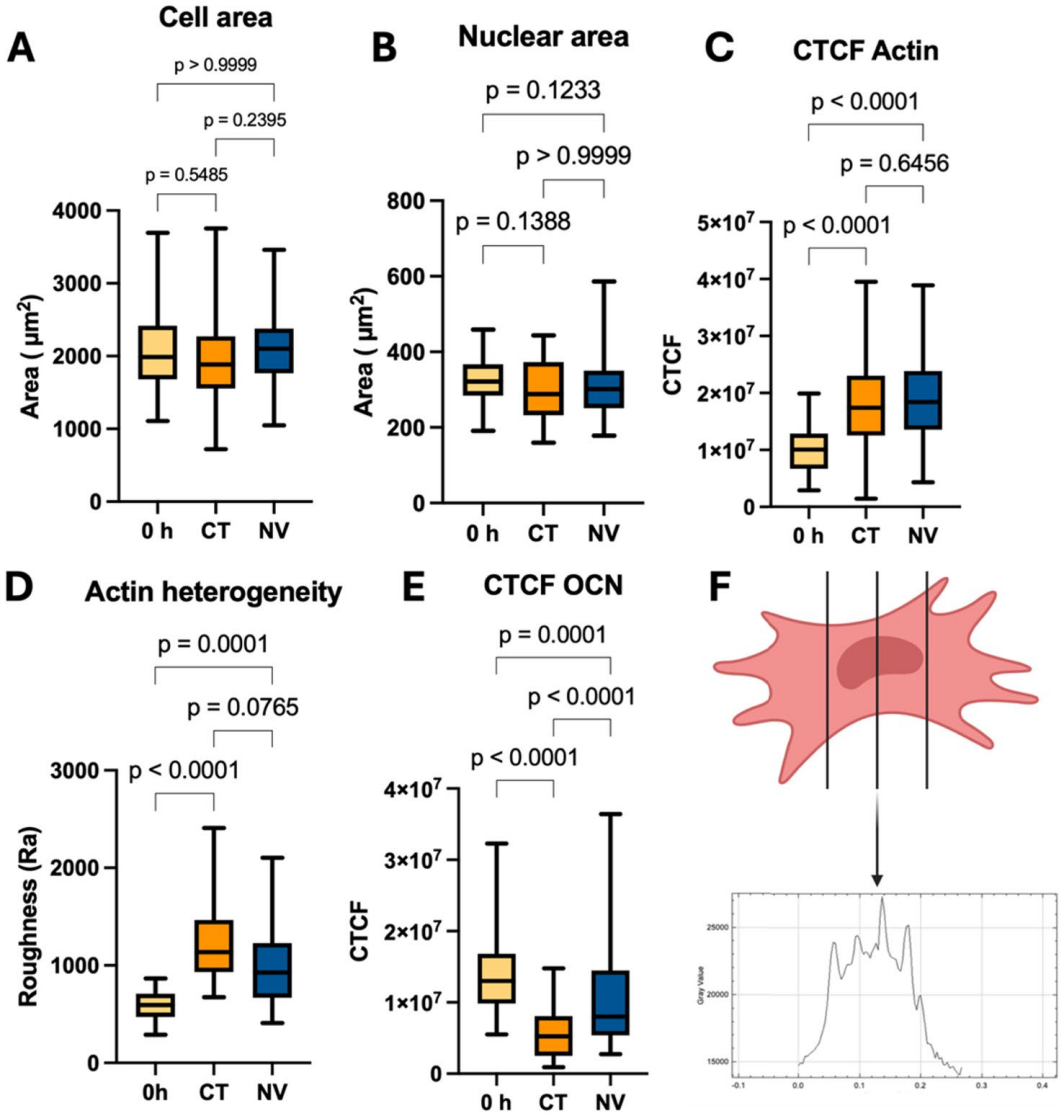
Immunocytochemical staining. (A) Fluorescent staining of Actin utilizing phalloidin showed no significant difference in the cell's area (*n* = 55–93 per condition). As well, (B) DAPI staining showed no significant changes in nuclear area (*n* = 55–93 per condition). Although there were no changes in cell or nuclear size, (C) actin's intensity increased in both control and nanovibrated groups indicating a greater density of Actin filaments (*n* = 55–93 per condition). Analyzing the actin's arrangement (D), looking for potential rearrangement of the cytoskeleton of the cells showed a significant increase in the heterogeneity of the cytoskeleton of both CT and NV groups compared to the control 0 h (*n* = 30 per condition). There was no significant difference between the CT and NV at the *p* < 0.05 level of significance presenting itself with a *p*‐value of 0.0765. (E) Assaying osteocalcin levels, both the CT and NV groups significantly dropped the expression of the late osteogenesis marker with CT presenting a significantly greater drop in OCN expression than the NV group (*n* = 55–93 per condition). (F) Heterogeneity of cells was determined by scanning Actin‐stained cells, as represented in Figure 4. Heterogeneity determined by strong deviations from mean gray value inside the cell.

Data were extracted from the images collected from this experiment utilizing ImageJ. Actin staining helped extract cell area, actin quantification, and actin arrangement. DAPI staining gave insights into nuclear size (indicative of cellular tension) and OCN staining helped quantify this protein's expression, as a marker of osteogenic response. It was noted that between 0 and 72 h and between mechanical treatment groups, there were no significant changes in cell area in their attached state (Figure 5A). There was, however, increased actin protein levels in the CT and NV groups compared to 0 h (Figure 5C).

An attempt to visualize and quantify changes in cytoskeletal arrangement was made, in order to explain the significant difference in stiffness between the two groups. Therefore, a metric for cytoskeletal heterogeneity was developed by defining a “roughness” (Figure 5F) for the imaged actin network [26] (Figure 5D). After applying this technique to the actin images, results showed that both CT and NV presented with increased actin heterogeneity compared to 0 h. with NV cells showing lower actin heterogeneity than CT cells (albeit non‐significantly, *p* = 0.0765). DAPI staining did not indicate any significant changes in the nuclear area between any of the three groups (Figure 5B). Osteocalcin staining indicated an initial decrease in its expression in both CT and NV groups compared to 0 h (Figure 5E). This is to be expected as OCN is typically reduced in expression as the cell re‐enters a proliferative state following seeding [28]. However, even at this early time point of 72 h, OCN staining was significantly higher in the NV group than CT.

## 7 Discussion

MG63's were mechanically treated utilizing a 1000‐Hz frequency, 30‐nm amplitude, sinusoidal vibration, previously identified to stimulate mesenchymal stem cell differentiation [15, 29]. This phenotypic change is now confirmed across multiple studies. However, mechanical changes of the cells when exposed to this treatment are still unclear. Current solutions, such as AFM, can only measure a small sample of cells, and the ability to measure large amounts of cells is impractical. As an example, in Campsie et al. (2019) [14], AFM was used to measure stiffness of collagen hydrogels seeded with cells while using the same mechanical treatment. However, measurements were limited to the bulk sample due to a lack of environmental control.

As previously explained, it was decided to use RT‐DC to mechanically profile single cells in large numbers, as seen in Figure 1. As this is a relatively new technique compared to the likes of AFM [21, 30], some protocol optimization to run these cells was required. The cells were seeded at 5000 cells/cm^2^, mechanically treated for 72 h, and tested at all three standard flow rates the equipment allows (0.08, 0.12, and 0.16 μL/s). However, the deformability of these cells at these flow rates was still relatively low and therefore was also tested at its maximum theoretical flow rate of 0.32 μL/s. This flow rate increased the deformation of both groups of cells while still showing the same behavior as the previous flow rates (Figure 2B). This flow rate increased the sensitivity of testing and allowed for easier gating of relevant cell populations. Once the test flow rate had been established, a time analysis of cell mechanical properties when exposed to the mechanical treatment was performed (Figure 2C). As seen at 24 h, no significant difference (*p* > 0.05) was observed, potentially due to the ongoing reconfiguration of the cytoskeleton. At 72 h, there was a significant (*p* < 0.001) decrease in deformability in the NV group compared to CT. This result was in line with the results obtained from the flow rate analysis testing. Finally, at 144 h, there was once again no significant (*p* > 0.05) difference between CT and NV. It was suspected that, due to the seeding density of the cells and their proliferation rate, the cells were increasingly confluent and in contact with each other. It was therefore postulated that those cell–cell interactions were greater mechanical stimulants than the nanovibration, or due to the multilayers of cells, the signal was attenuated and masked by cell‐to‐cell interactions. This would be congruent with the study conducted by Collins et al. (2016) [31] where they describe extensive studies on substrate rigidity impact on cells but not E‐cadherin rigidity sensing. E‐cadherins, being a cell‐to‐cell adhesion molecule [32], have been demonstrated to be able to sense rigidity and induce cytoskeletal changes, which could help explain the cell's mechanical property's changes.

The lack of a significant deformability difference at 24 h may reflect a lag in the emergence of a stable whole‐cell mechanical phenotype. RT‐DC is highly sensitive to cytoskeletal state, but detectable shifts in deformability are expected only once early mechanotransductive and transcriptional events have progressed to appreciable remodeling of the actin cortex/cytoskeleton [21]. This delayed mechanical divergence is consistent with prior MSC literature showing that early adhesion/spreading responses can occur within 24 h, whereas more mature cytoskeletal tension‐associated features such as prominent F‐actin stress fibers, nuclear expansion, and Lamin A upregulation only become significant around 72 h; similarly, integrin/FAK‐dependent adhesion signaling is required upstream of osteogenic commitment [33]. This data also highlights the need to develop new metrics for cytoskeletal configuration (e.g., switches between cortex and stress fiber configurations) with potential to correlate with deformability values. Additionally, the high‐throughput, high sensitivity nature of RT‐DC raises the question of whether it could detect changes earlier than would be possible by cytoskeletal imaging, where sample sizes are typically much smaller.

To understand whether the mechanical treatment was influencing the proliferation rate of MG63s and, as a result, causing less cell‐to‐cell interactions due to the reduced number of cells in NV, an MTT assay was carried out. Further, cells were seeded at 500, 1000, and 5000 cells/cm^2^ to determine whether a lower seeding density could be utilized, thus allowing for longer studies before reaching confluency. Results from the MTT assay (Figure 3A) showed no statistical differences between CT and NV proliferation rates in both seeding densities. Therefore, NV shows no effect in proliferation for this cell type. Therefore, mechanical differences between CT and NV can be inferred as being caused by response to the mechanical signal alone. In parallel, cells were also tested for their deformability at their different seeding densities (Figure 3B). It is important to reiterate that the 0 h CT measurement involved a seeding density of 5000 cells/cm^2^ as the number of cells at other seeding densities was too low to perform RT‐DC. Results showed a significantly (*p* < 0.0001) lower deformability following NV compared to CT at all three seeding densities. This indicates that RT‐DC is sensitive enough to distinguish population‐level mechanical cellular changes caused by nanovibration, against a background of larger changes related to seeding density and cell confluency. As predicted, both CT and NV increase in deformability as the seeding density increases. This indicates that cells naturally change their mechanical properties simply by having contact with other cells as demonstrated by Collins et al. (2016) [31]. It also showed that the same response to treatment is observed at lower seeding densities. Therefore, further experiments were seeded at 1000 cells/cm^2^ as it established the middle ground between the quantity of cells and longevity of the experiment.

Once test parameters were optimized, morphology was assessed in an attempt to justify the changes in deformability at 72 h. The nucleus and actin cytoskeleton of cells were stained using immunocytochemistry (ICC) as seen in Figure 4. Furthermore, osteocalcin (OCN) as an osteoblast marker was measured [15]. At 72 h, there were no significant (*p* > 0.05) cell area differences between CT and NV (Figure 5A). There was, however, increased actin levels in the CT and NV groups compared to 0 h (Figure 5C). This indicates the cells are polymerizing actin filaments without increasing in size nor, changing their proliferation rate as previously described in Figure 3A. With no significant difference in actin between the two groups, it was hypothesized that different arrangements of actin were causing the deformability differences. Profilometry of the actin staining was used to measure the arithmetic mean difference of the absolute value differences of the profile deviations from the mean line of the profile as seen in Figure 5F. The principle assumes that increased actin staining intensity is proportional to more or thicker actin fibers in that particular location, that is, a stress fiber would be visible in an intensity profile as a peak compared to the average profile actin intensity. Therefore, a more fibrous cytoskeleton that favors stress fiber formation will present higher roughness values when a profile of actin intensity is taken across the cell. As seen in Figure 5D, there were no significant (*p* > 0.05) differences between CT and NV when comparing the mean roughness between all three groups present. Further statistical testing can be carried out between CT and NV as testing between control vs. treatment; however, care needs to be taken when interpreting these results as significance was already rejected at group level. When conducting a Mann–Whitney test between the two, there was statistical significance between CT and NV (*p* = 0.0162). These data could show that both CT and NV modulate their cytoskeleton toward a more fibrous arrangement after 72 h in culture. The results at 72 h indicate that the balance between stress fiber and actin cortex formation may be important for the cellular deformability when detached. We note that the trend of actin roughness between all three groups, matches the trends seen in deformability. As previously stated, MG63s preserve the capability of expressing osteogenic markers such as OCN, a marker indicating late‐stage osteoblasts [34]. Osteocalcin staining offered some insights into cell expressional behavior. Both groups significantly (*p* < 0.0001) reduced expression levels compared to 0 h; however, NV OCN levels were significantly higher (p < 0.0001) than CT. A study of chemically stimulated MG63s showed that basal secretion of OCN is minimal/not‐measurable, but the cells are capable of increasing this expression when osteogenically stimulated [35]. As shown by the MTT assay, there was no difference between the proliferation of the CT and NV groups. This could indicate that while MG63s innately reduce OCN levels during culture, mechanical treatment counteracts this, encouraging osteogenesis. The authors do acknowledge that changes in the MG63s may not directly translate in changes in primary osteoblasts.

On the point of comparability of RT‐DC data and methods such as AFM, it is clear that there are significant process differences, that is, the requirement to detach cells from the growth surface. It is entirely plausible that cytoskeletal changes result from this process, impacting the absolute mechanical properties of the cells. For this reason, we consider this technique's value to be in the relative measurement of groups, rather than as a direct comparator for the elastic moduli values obtained via AFM. However, it is reassuring that previous studies demonstrate that trends seen in AFM are also seen with RT‐DC, indicating that some mechanical features are conserved upon detachment [36].

In conclusion, we report on a mechanical treatment known as nanovibration and its impact on deformability, as measured by RT‐DC. This treatment consists of the administration of mechanical oscillations of 1000‐Hz frequency and 30‐nm amplitude provided by a calibrated vibration device [13, 14, 15, 37]. The optimal duration of the mechanical treatment in a continuous manner was determined to be 72 h to maximize changes in deformability of MG63 cells. Furthermore, the optimal seeding density of this cell line for a treatment period of 72 h is 1000 cells/cm^2^ to minimize the confounding effect of cell‐to‐cell interactions in higher density culture. In addition, the cells demonstrated morphological changes in their cytoskeleton together with changes in osteocalcin expression. This indicates the cells were capable of sensing the mechanical stimulation and responding to it with trends matching those seen in deformability, that is, higher levels of osteocalcin related to increased actin homogeneity and lower deformability. This study reached the limit of value that MG63s are capable of producing as an osteogenic model; however, this study provides a first indication that RT‐DC may be relevant as a tool to probe the mechanical state of cells. It is crucial to understand cell mechanical behavior during osteogenesis for the treatment of osteoporosis. This study lays the groundwork for more extensive testing on MSCs and to get us one step closer to understand how we can mechanically treat and analyze these cells during osteogenesis, while going beyond traditional protein‐based biomarkers. It is paramount that model studies are executed to validate methodologies and project planning prior advancing to costly and harder to source cells such as human bone marrow‐derived MSCs.

## Author Contributions

R.P.P.S., P.G.C. and M.J. conceived and designed the research R.P.P.S. performed the research and acquired the data, R.P.P.S., P.G.C., and M. Jimenez analyzed and interpreted the data. R.P.P.Sousa, P.G.C., M.J., M.J.D., and S.R. were involved with analysis and interpretation of data. All authors were involved in drafting and revising the manuscript.

## Funding

This work was supported by UKRI | Engineering and Physical Sciences Research Council (SRC), EP/W524670/1. Royal Academy of Engineering (RAENG), RF\201718\1741.

## Conflicts of Interest

The authors declare no conflicts of interest.

## Data Availability

The data that support the findings of this study are openly available in https://doi.org/10.15129/d7005a63‐03c9‐4920‐bdeb‐0a1cd98fca36. RT‐DC data sets can be made available on reasonable request.
